# Compliance with a Healthful Plant-Based Diet Is Associated with Kidney Function in Patients with Autosomal Dominant Polycystic Kidney Disease

**DOI:** 10.3390/nu16162749

**Published:** 2024-08-17

**Authors:** Sumin Heo, Miyeun Han, Hyunjin Ryu, Eunjeong Kang, Minsang Kim, Curie Ahn, Soo Jin Yang, Kook-Hwan Oh

**Affiliations:** 1 Department of Food and Nutrition, Seoul Women’s University, Seoul 01797, Republic of Korea; 2 Department of Internal Medicine, National Medical Center, Seoul 04564, Republic of Korea; 3 Department of Internal Medicine, Seoul National University Hospital, Seoul 03080, Republic of Korea

**Keywords:** autosomal dominant polycystic kidney disease, neutrophil-to-lymphocyte ratio, plant-based diet, plant protein, platelet-to-lymphocyte ratio

## Abstract

Autosomal dominant polycystic kidney disease (ADPKD) is a genetic kidney disorder with multiple cyst formation that progresses to chronic kidney disease (CKD) and end-stage kidney disease. Plant-based diets have attracted considerable attention because they may prevent CKD development. This study investigated whether adherence to a plant-based diet is associated with kidney function in patients with ADPKD. The overall plant-based diet index (PDI), healthful PDI (hPDI), and unhealthful PDI (uPDI) were calculated using dietary intake data. Among 106 ADPKD patients, 37 (34.91%) were classified as having advanced CKD (eGFR < 60 mL/min/1.73 m^2^). The overall PDI and hPDI were lower, but the uPDI was higher in patients with advanced CKD than in those with early CKD. The hPDI was negatively correlated with the neutrophil-to-lymphocyte ratio and platelet-to-lymphocyte ratio. Moreover, the hPDI was inversely associated with advanced CKD [odds ratio (OR): 0.117 (95% confidence interval (CI): 0.039–0.351), *p* < 0.001], and the uPDI was positively associated with advanced CKD [OR: 8.450 (95% CI: 2.810–25.409), *p* < 0.001]. The findings of the current study demonstrate that greater adherence to a healthful plant-based diet is associated with improved kidney function in ADPKD patients.

## 1. Introduction

Autosomal dominant polycystic kidney disease (ADPKD) is a hereditary kidney disorder caused by mutations in the *PKD1* or *PKD2* genes encoding polycystin 1 (PC1) or PC2 and is characterized by the growth of excessive cysts in the kidneys [1]. The estimated prevalence of ADPKD is approximately 3.96 to 9.3 cases in 10,000 individuals [2,3], and ADPKD accounts for 5–10% of end-stage kidney disease (ESKD) cases [4]. Tolvaptan, a selective vasopressin V2 receptor antagonist, has been used for the pharmacological treatment of ADPKD [5]. In addition, lifestyle modifications, including dietary management, are necessary to delay the progression of chronic kidney disease (CKD) and prevent complications like hypertension [6].

The dietary guidelines for patients with ADPKD suggest a healthy diet that limits sodium and alcohol intake [6], and clinicians often apply dietary guidelines for CKD to manage symptoms and delay the progression of CKD in patients with ADPKD. General nutritional guidelines for CKD include reducing dietary protein and sodium intake and managing the dietary intake of calcium, phosphorus, and potassium to maintain these serum concentrations within the normal range [7]. However, strict protein restriction is not always recommended, and individualized dietary strategies should be applied to patients with ADPKD [7].

In addition to the nutrient-focused recommendation, recent evidence suggests that dietary patterns such as the Mediterranean diet and a diet with increased fruits and vegetables may improve the lipid profile or reduce body weight, blood pressure, and net acid production in CKD patients [7]. Moreover, caloric restriction, intermittent fasting, time-restricted feeding, and a ketogenic diet are suggested as potential dietary interventions for ADPKD owing to the metabolic benefits of these dietary patterns [8,9]. However, the supporting evidence is currently insufficient to draw a definitive conclusion or make a recommendation.

A plant-based diet is a dietary pattern with lower portions of animal foods and higher portions of plant foods such as whole grains, fruits, vegetables, nuts, legumes, and seeds [10]. There is supporting evidence regarding its beneficial effects on chronic diseases such as cardiovascular diseases and diabetes [11,12]. The reasons accounting for the health effects of plant-based diets are assumed to be related to the substantial amounts of dietary fiber, antioxidants, and phytochemicals found in plant foods [10,13]. Although high amounts of plant foods were not recommended for patients with CKD due to the risk of a high dietary intake of potassium, recent investigations have demonstrated that adherence to a plant-based diet as a whole dietary pattern is associated with the prevention of CKD and its complications [14,15]. While supporting evidence regarding the beneficial effects of plant-based diets in CKD has been accumulating, the association between plant-based diets and kidney function in patients with ADPKD has not yet been investigated.

Therefore, this study aimed to test the hypothesis that adherence to a plant-based diet is associated with kidney function in patients with ADPKD. To test this hypothesis, three types of plant-based diet index (PDI) scores were calculated from dietary intake data, and the association between the PDI scores and kidney function was analyzed in patients with ADPKD.

## 2. Materials and Methods

### 2.1. Study Participants

**Figure 1:**
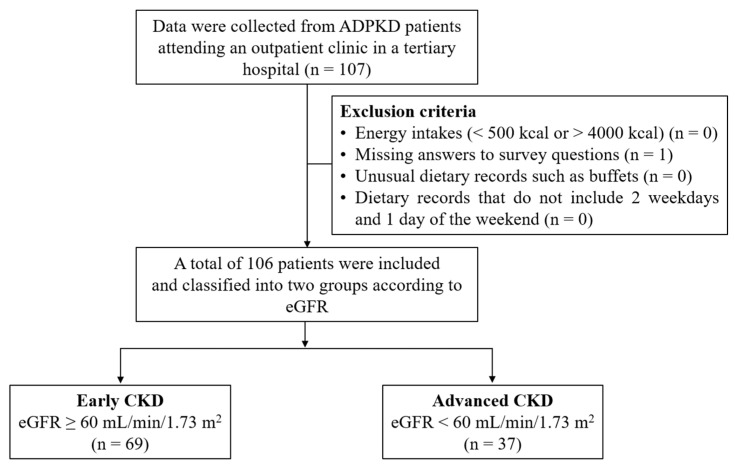
The flow diagram for study participants. ADPKD, autosomal dominant polycystic kidney disease; CKD, chronic kidney disease; eGFR, estimated glomerular filtration rate.

Participants were recruited from patients attending the outpatient clinic of a tertiary hospital between April 2017 and March 2018. The inclusion criterion was adults (age > 18 years) diagnosed with ADPKD who were not undergoing dialysis or had not undergone kidney transplantation. The exclusion criteria included participants with an unrealistic energy intake (<500 kcal or >4000 kcal), missing answers to survey questions, unusual dietary records such as buffets, and dietary records that did not include 2 weekdays or 1 weekend day. A total of 107 participants completed the questionnaires; 1 was excluded because of missing answers to the survey questions. Data on age, sex, anthropometry (height, body weight, and body mass index), disease history (anemia, diabetes, dyslipidemia, gout, hypertension, and hyperuricemia), blood pressure (systolic blood pressure and diastolic blood pressure), and biochemical parameters related to kidney function were also collected. During face-to-face interviews, trained dietitians completed 3-day food records on 2 weekdays and 1 weekend day. A total of 106 patients (53 men and 53 women) were classified according to the estimated glomerular filtration rate (eGFR) into early CKD (≥60 mL/min/1.73 m^2^; n = 69) and advanced CKD (<60 mL/min/1.73 m^2^; n = 37) groups. Figure 1 presents a flowchart of the study participants. This study protocol was approved by the Institutional Review Board of Seoul National University Hospital (H-1702-107-833), and all participants provided written informed consent. All experiments were performed in accordance with the ethical standards of The Helsinki Declaration.

### 2.2. Clinical Variables

Blood samples were obtained to analyze the biochemical parameters related to kidney function. Data on erythrocyte sedimentation rate (ESR), neutrophil-to-lymphocyte ratio (NLR), platelet-to-lymphocyte ratio (PLR), serum albumin, hemoglobin, hematocrit, eGFR, blood urea nitrogen (BUN), serum creatinine (Scr), serum calcium, serum phosphorus, serum potassium, and serum sodium were collected. The eGFR was calculated using the Chronic Kidney Disease Epidemiology Collaboration (CKD-EPI) equation (CKD-EPI equation = 141 × min (Scr/κ, 1)^α^ × max (Scr/κ, 1)^−1.209^ × 0.993^Age^ × 1.018 [if female]; κ is 0.7 for females and 0.9 for males, α is −0.329 for females and −0.411 for males) [16].

### 2.3. Dietary Assessment and PDIs

Dietary intake data were obtained from 3-day food records. Trained dietitians completed dietary records for 2 weekdays and 1 weekend day during face-to-face interviews with the study participants. Dietary intake data, including the amount of food intake, specific recipes, ingredients of foods, and names of commercial products, were collected. The 3-day food records were checked using food models (Mirage Replica Inc., Incheon, Republic of Korea) and analyzed using the CAN-Pro 6.0 program (The Korean Nutrition Society, Seoul, Republic of Korea). The three types of PDI (overall PDI, a healthful PDI (hPDI), and an unhealthful PDI (uPDI)) were calculated according to the PDI scoring system [17]. The 18 food groups were categorized into healthy plant foods, less healthy plant foods, and animal foods. Healthy plant food groups consist of whole grains, fruits, vegetables, nuts, legumes, vegetable oils, and tea and coffee. The less healthy plant food groups consist of fruit juices, refined grains, potatoes, sugar-sweetened beverages, and sweets and desserts. The animal food groups comprised animal fat, dairy, eggs, fish or seafood, meat, and miscellaneous animal-based foods. The amount of food for each food group was converted to a serving size per day. One to five points were assigned according to the quintile of serving size for daily food consumption. The overall PDI was given a positive score for the healthy and less healthy plant food groups and a reverse score for the animal food group. The hPDI was calculated by only giving a positive score for the healthy plant food group and a reverse score for the other groups. The uPDI was also given a reverse score to all groups, except for the less healthy plant food group. Table 1 shows the scoring system for the three types of PDIs.

### 2.4. Statistical Analysis

The primary exposures were the PDIs and dietary intake of fiber, protein, animal protein, and plant protein. The outcomes were changes in blood pressure, ESR, NLR, PLR, serum albumin, hemoglobin, hematocrit, eGFR, BUN, Scr, serum calcium, serum phosphorus, serum potassium, and serum sodium.

The general characteristics, clinical variables, and dietary intake of the study participants were demonstrated as means and standard deviations for quantitative variables or counts and percentages for qualitative variables. Individuals were classified according to the eGFR cut-off value (60 mL/min/1.73 m^2^). Differences in general characteristics, clinical variables, and dietary intake were evaluated. Student’s *t*-tests were applied to continuous variables that followed a normal distribution. If the data did not follow a normal distribution, the Mann–Whitney U test was used for continuous variables. Categorical variables were analyzed using either the chi-square test or Fisher’s exact test. Participants were categorized by the medians of their PDIs or the cut-off value (0.8 g/kg body weight/day) of dietary protein intake, and clinical variables and dietary intake were compared using Student’s *t*-test or Mann–Whitney U test. Pearson’s correlation test was employed to assess the correlation between nutritional characteristics (PDIs, dietary fiber, and dietary protein) and blood pressure, eGFR, and biochemical parameters. The associations between PDIs, dietary fiber intake, and protein intake with advanced CKD were analyzed using logistic regression analysis unadjusted or adjusted for age, sex, and energy intake. Statistical analyses were considered significant if *p* < 0.05 and were conducted using IBM SPSS Statistics software (version 26.0; IBM Corp., Armonk, NY, USA).

## 3. Results

### 3.1. General Characteristics of the Study Participants by eGFR

Table 2 outlines the general characteristics of the study participants according to their eGFR. Participants were considered to have advanced CKD when the eGFR was below 60 mL/min/1.73 m^2^. Of the 106 patients with ADPKD, 37 (34.91%) were classified as having advanced CKD. Participants with advanced CKD were older and more often diagnosed with anemia, dyslipidemia, or hypertension. Their serum albumin, hemoglobin, hematocrit, and eGFR levels were lower; however, their BUN, Scr, and serum potassium concentrations were greater.

### 3.2. PDIs According to the eGFR and the Correlation between PDIs and Kidney Function

**Figure 2:**
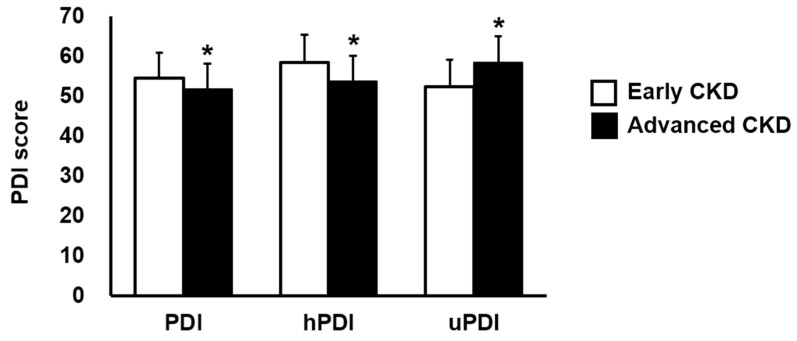
Three types of plant-based diet indices (PDIs) by the levels of estimated glomerular filtration rate (eGFR). Data are demonstrated as means ± standard deviations. Student’s *t*-test was applied for variables with a normal distribution. However, if normality was not satisfied, the Mann–Whitney U test was used. * *p* < 0.05. CKD, chronic kidney disease; hPDI, healthful plant-based diet index; uPDI, unhealthful plant-based diet index.

Figure 2 shows the PDIs by eGFR in the participants. The overall PDI and hPDI were significantly lower but the uPDI was significantly greater in patients with advanced CKD.

Pearson’s correlation analyses were performed to explore the correlation between PDIs and clinical variables. In addition, correlations were determined among the inflammation-related parameters ESR, NLR, and PLR. The ESR was positively correlated with the NLR (*r* = 0.477, *p* < 0.001) and the PLR (*r* = 0.413, *p* = 0.002). The overall PDI was negatively correlated with Scr concentration (Table 3). The hPDI was negatively correlated with NLR, PLR, BUN, and Scr; however, it was positively correlated with eGFR. The uPDI was negatively correlated with eGFR and positively correlated with BUN and serum concentrations of creatinine, potassium, and sodium.

### 3.3. Associations between PDIs and Kidney Function

Participants were stratified by median values of PDIs, and clinical variables related to kidney function were compared between the low- and high-PDI groups. The median values of the overall PDI, hPDI, and uPDI were 54, 57, and 54, respectively. There were no differences in the clinical variables assessed between the low and high groups of the overall PDI (Table 4).

However, NLR, PLR, BUN, and Scr levels were lower in the high-hPDI group, and eGFR was higher in the high-hPDI group than in the low-hPDI group. Moreover, participants in the high-uPDI group had a lower eGFR and higher BUN, Scr, and serum potassium concentrations than those in the low-uPDI group. In the logistic regression model, the high hPDI was inversely associated with advanced CKD versus the low hPDI [OR: 0.117 (95% CI: 0.039–0.351), *p* < 0.001] (Table 5). In contrast, the high uPDI was positively associated with advanced CKD versus the low uPDI [OR: 8.450 (95% CI: 2.810–25.409), *p* < 0.001].

### 3.4. Dietary Intakes of Study Participants According to the Median Values of PDIs

The dietary intakes of the study participants were compared between the low- and high-PDI groups. The dietary intakes of carbohydrates, fiber, and vitamin E were higher in participants in the high-overall-PDI group (Table 6). Dietary intakes of fiber and vitamin C were higher and the dietary intake of animal protein was lower in participants in the high-hPDI group. Participants in the high-uPDI group had a lower dietary intake of fiber, protein, animal protein, fat, polyunsaturated fatty acids (PUFAs), omega-3 PUFAs, vitamins E, B_1_, B_2_, niacin, B_6_, folate, phosphorus, sodium, and potassium than those in the low-uPDI group.

**Figure 3:**
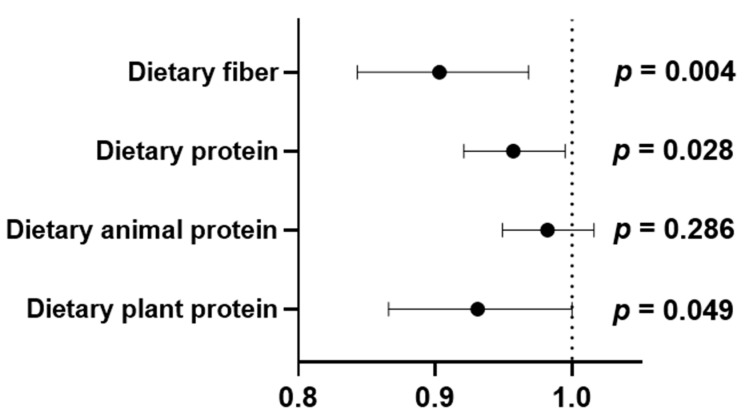
Associations of dietary fiber and protein intakes with advanced chronic kidney disease (CKD). Odds ratios with 95% confidence intervals (CIs) for advanced CKD adjusted for age, sex, and energy intake by logistics regression analysis. Statistical significance was considered as *p* < 0.05.

The frequency of reduced protein intake (< 0.8 g/kg body weight/day) by the medians of the PDIs is summarized in Table 7. The frequency of reduced protein intake differed significantly between the low and high groups for all the PDIs. Specifically, there was a higher frequency of reduced protein intake in the low groups of overall PDI and hPDI and in the high uPDI group. Dietary fiber was the only nutrient that differed between the low and high groups of all PDIs, and the amount and type of dietary protein are important issues to consider in the nutritional management of ADPKD. Therefore, the correlations between the dietary intake of fiber, protein, animal protein, and plant protein and clinical variables were analyzed (Table 8). Dietary fiber intake was negatively correlated with NLR, BUN, and Scr levels. The dietary intake of fiber, protein, and animal protein was positively correlated with eGFR. In addition to its correlation with eGFR, dietary protein intake was negatively correlated with BUN and Scr levels. Dietary plant protein was negatively correlated with NLR, PLR, and BUN and positively correlated with hemoglobin. Moreover, the dietary intakes of fiber, protein, and plant proteins were inversely associated with advanced CKD (Figure 3 and Supplementary Table S1).

Blood pressure, eGFR, and biochemical parameters according to the median values of PDIs and the presence of a reduced protein intake are summarized in Supplementary Tables S2–S4. In the high-PDI group, the eGFR was lower, and serum concentrations of creatinine, calcium, and sodium were higher in the reduced protein intake group than in the normal protein intake group (Supplementary Table S2). Similarly, regarding hPDI and protein intake, in the high-hPDI group, the serum concentrations of calcium, phosphorus, and sodium were significantly greater in the reduced protein intake group than in the normal protein intake group (Supplementary Table S3). The clinical variables according to the median values of the uPDI and the presence of a reduced protein intake were also compared, and the comparison results showed that, in the low-uPDI group, the NLR was significantly higher in the reduced protein intake group than in the normal protein intake group (Supplementary Table S4).

## 4. Discussion

This study investigated the association between PDIs and advanced CKD in patients with ADPKD. Among the scored PDIs, the overall PDI and hPDI were lower; however, the uPDI was significantly higher in patients with advanced CKD. Moreover, the PDIs were significantly associated with advanced CKD. In particular, the hPDI and dietary intake of plant proteins were negatively correlated with NLR and PLR and inversely associated with advanced CKD in patients with ADPKD.

ADPKD is a genetic kidney disorder characterized by increases in the number and volume of kidney cysts [4]. Patients with ADPKD experience a decline in kidney function and subsequent CKD progression, often leading to ESKD [4]. Therefore, intensive management is required for patients with ADPKD to maintain kidney function and delay CKD progression. In this study, 34.91% of participants were classified as having advanced CKD. The proportion of patients with advanced CKD in ADPKD patients would be higher than the current estimate because the current study included only non-dialysis patients. Dietary guidelines for ADPKD suggest a balanced healthy diet that restricts sodium and alcohol consumption [6]. Once patients with ADPKD are diagnosed with CKD, they are often recommended to follow the dietary guidelines for CKD. However, owing to its complexity, more individualized and specific dietary strategies should be considered to manage ADPKD.

A plant-based diet contains a high proportion of plant foods such as whole grains, fruits, vegetables, nuts, legumes, and seeds [10]. Traditionally, plant-based diets have not been considered for managing CKD and ADPKD. However, recent evidence suggests that a plant-based diet or plant foods may be helpful for preserving and improving kidney function [14,15]. A prospective analysis of middle-aged adults in the Atherosclerosis Risk in Communities study revealed that a healthy plant-based diet was linked to a reduced risk of CKD [14]. Another cross-sectional analysis of the Ravansar noncommunicable diseases cohort study reported an inverse association between PDI and CKD [18]. In addition, a recent report analyzing the data from the UK Biobank Study showed that plant protein intake was inversely associated with the risk of incident CKD [19]. Although the evidence regarding plant-based diets and CKD is consistent, reports on ADPKD are not yet available.

In the cross-sectional analysis of patients with ADPKD, we demonstrated a lower adherence to an overall plant-based diet and a healthful plant-based diet and higher adherence to an unhealthful plant-based diet in CKD patients with ADPKD. In addition to the positive correlation of eGFR and negative correlation of BUN and Scr with adherence to a healthful plant-based diet, the hPDI was negatively correlated with NLR and PLR. Recently, the NLR and PLR have been suggested as peripheral blood inflammatory indices [20,21]. In previous analyses, both NLR and PLR had a positive correlation with high-sensitivity C-reactive protein concentrations [20,21]. The current study demonstrated that NLR and PLR had a positive correlation with ESR, an inflammatory marker. These findings indicate that a healthful plant-based diet may help to reduce inflammation in ADPKD patients.

Furthermore, greater adherence to a healthful plant-based diet was inversely associated with advanced CKD in patients with ADPKD. A healthy plant-based diet is considered as beneficial for kidney function due to its lower burden on the kidney and its antioxidant and anti-inflammatory effects [15,22]. A healthy plant-based diet has a low acid load, low phosphorus bioavailability, and low uremic toxin production [15]. In the current analysis, participants who were more likely to adhere to a healthful plant-based diet did not have hyperphosphatemia or hyperkalemia. However, higher compliance with an unhealthful plant-based diet was associated with impaired kidney function, as shown by lower eGFR and higher BUN and Scr levels, which were accompanied by higher concentrations of serum potassium compared with participants with lower compliance with an unhealthful plant-based diet. The strong association between adherence to an unhealthful plant-based diet and kidney function may result from an unhealthy diet that includes high portions of refined foods, added sugars, and processed foods, and low portions of nutritious foods. In this study, the dietary intake of beneficial nutrients with anti-oxidative and anti-inflammatory effects, including fiber, protein, PUFAs, omega-3 PUFAs, and vitamin E [15,22], was low in patients with a higher adherence to an unhealthful plant-based diet, which may deteriorate kidney function in patients with ADPKD.

Dietary fiber is a beneficial nutrient in plant-based diets. It alters the gut microbiota to reduce uremic toxin formation, helps to excrete excessive or unnecessary materials, and exerts antioxidative and anti-inflammatory effects [23,24,25,26]. In the current study, the dietary fiber intake differed between the low- and high groups of all PDIs and was correlated with kidney function. In addition, the frequency of reduced protein intake was lower in the high-PDI and -hPDI groups, and dietary protein intake was related to kidney function, with a positive correlation with eGFR and a negative correlation with BUN and Scr levels. An interesting finding regarding plant-based diets and protein intake was that participants with greater adherence to plant-based diets and reduced protein intake had impaired kidney function. In general, protein restriction is recommended for CKD, regardless of the consideration of plant-based diets, to prevent progression to ESKD [7,27]. However, a study of ADPKD reported no relationship between protein intake and CKD progression [28]. A recent review suggested an adequate dietary protein intake of no more than 1.3 g/kg body weight/day when the GFR is ≥30 mL/min/1.73 m^2^, and 0.8–1.0 g protein/kg body weight/day when the GFR is <30 mL/min/1.73 m^2^ [7,29]. Based on this evidence and the findings from the current study, protein restriction may not be guided for the dietary management of ADPKD, and adequate protein intake ranging from 0.8 to 1.0 g protein/kg body weight/day may need to be considered when a healthful plant-based diet is applied to patients with ADPKD.

In addition to dietary fiber and protein in plant-based diets, the source of dietary protein has been an issue of great interest in the dietary management of CKD and ADPKD. Traditional recommendations regarding dietary protein sources include those with high bioavailability, which are generally animal foods [30]. However, recent reports on CKD suggest that a higher plant protein intake is related to a reduced risk of CKD [19,31]. According to the current analyses of ADPKD patients, dietary plant proteins were negatively correlated with the peripheral inflammatory markers NLR and PLR and with BUN, and were inversely associated with advanced CKD. These findings support previous evidence regarding the relationship of dietary plant proteins with kidney function and the risk of incident CKD. However, correlation analysis also revealed a positive correlation between dietary animal protein and eGFR, implying that an adequate protein intake may be more important than the type of dietary protein. As these implications do not have direct evidence, well-designed, large-scale, comparative studies should be conducted to establish dietary guidelines on the amount and type of dietary proteins for ADPKD.

The current study reported that higher compliance with a healthful plant-based diet had an inverse association with advanced CKD in patients with ADPKD. Moreover, higher adherence to a healthful plant-based diet and a dietary plant protein intake were negatively correlated with peripheral inflammatory parameters NLR and PLR, and inversely associated with advanced CKD in ADPKD patients. Despite these meaningful findings regarding the dietary management of ADPKD patients, this study had limitations. First, the sample size was comparatively small, which granted a large-scale study. Second, the current analysis did not investigate cause–effect relationships. Third, microalbuminuria or total kidney volume was not considered in the analysis. Extended analyses including these factors would provide valuable evidence considering the progression of CKD and ADPKD. Fourth, the current investigation was based on a single-center cross-sectional study. A large-scale, global multi-center study should be conducted to validate the current findings and establish dietary guidelines for patients with ADPKD. Additionally, further studies to determine the dietary pattern, as well as the amount and type of dietary protein for ADPKD patients, are recommended.

## Figures and Tables

**Table 1:** The scoring system of the three types of plant-based diet indices (PDIs).

Plant Food Groups		PDI	hPDI	uPDI
**Healthy**				
Whole grains	Whole-grain breakfast cereal, other cooked breakfast cereal, cooked oatmeal, dark bread, brown rice, other grains, bran, wheat germ, popcorn	Positive	Positive	Reverse
Fruits	Raisins or grapes, prunes, bananas, cantaloupe, watermelon, fresh apples or pears, oranges, grapefruit, strawberries, blueberries, peaches or apricots or plums	Positive	Positive	Reverse
Vegetables	Tomatoes, tomato juice, tomato sauce, broccoli, cabbage, cauliflower, Brussels sprouts, carrots, mixed vegetables, yellow or winter squash, eggplant or zucchini, yams or sweet potatoes, spinach cooked, spinach raw, kale or mustard or chard greens, iceberg or head lettuce, romaine or leaf lettuce, celery, mushrooms, beets, alfalfa sprouts, garlic, corn	Positive	Positive	Reverse
Nuts	Nuts, peanut butter	Positive	Positive	Reverse
Legumes	String beans, tofu or soybeans, beans or lentils, peas or lima beans	Positive	Positive	Reverse
Vegetable oils	Oil-based salad dressing, vegetable oil used for cooking	Positive	Positive	Reverse
Tea and coffee	Tea, coffee, decaffeinated coffee	Positive	Positive	Reverse
**Less healthy**				
Fruit juices	Apple cider (non-alcoholic) or juice, orange juice, grapefruit juice, other fruit juice	Positive	Reverse	Positive
Refined grains	Refined-grain breakfast cereal, white bread, English muffins or bagels or rolls, muffins or biscuits, white rice, pancakes or waffles, crackers, pasta	Positive	Reverse	Positive
Potatoes	French fries, baked or mashed potatoes, potato or corn chips	Positive	Reverse	Positive
Sugar-sweetened beverages	Colas with caffeine and sugar, colas without caffeine but with sugar, other carbonated beverages with sugar, non-carbonated fruit drinks with sugar	Positive	Reverse	Positive
Sweets and desserts	Chocolates, candy bars, candy without chocolate, cookies (home-baked and ready-made), brownies, doughnuts, cake (home-baked and ready-made), sweet rolls (home-baked and ready-made), pies (home-baked and ready-made), jams or jellies or preserves or syrup or honey	Positive	Reverse	Positive
**Animal food groups**		**PDI**	**hPDI**	**uPDI**
Animal fat	Butter added to food, butter or lard used for cooking	Reverse	Reverse	Reverse
Dairy	Skimmed low-fat milk, whole milk, cream, sour cream, sherbet, ice cream, yogurt, cottage or ricotta cheese, cream cheese, other cheese	Reverse	Reverse	Reverse
Egg	Eggs	Reverse	Reverse	Reverse
Fish or seafood	Canned tuna, dark meat fish, other fish, shrimp or lobster or scallops	Reverse	Reverse	Reverse
Meat	Chicken or turkey with skin, chicken or turkey without skin, bacon, hot dogs, processed meats, liver, hamburger, beef or pork or lamb mixed dish, beef or pork or lamb main dish	Reverse	Reverse	Reverse
Miscellaneous animal-based foods	Pizza, chowder or cream soup, mayonnaise or other creamy salad dressing	Reverse	Reverse	Reverse

hPDI, healthful plant-based diet index; uPDI, unhealthful plant-based diet index.

**Table 2:** General characteristics of the participants by the levels of estimated glomerular filtration rate (eGFR).

	Early CKD (eGFR ≥ 60 mL/min/1.73 m^2^; n = 69)	Advanced CKD (eGFR < 60 mL/min/1.73 m^2^; n = 37)	*p*
Age (y)	49.80 ± 12.39	60.00 ± 9.10	**<0.001**
Sex (M/F)	33/36	20/17	0.541
Height (cm)	164.37 ± 8.70	165.23 ± 8.02	0.618
Body weight (kg)	63.08 ± 11.49	62.51 ± 10.91	0.805
BMI (kg/m^2^)	23.25 ± 3.06	22.76 ± 2.81	0.428
Anemia, n (%)	5 (7.2)	12 (32.4)	**0.001**
Diabetes, n (%)	2 (2.9)	3 (8.1)	0.340
Dyslipidemia, n (%)	20 (29.0)	20 (54.1)	**0.011**
Gout, n (%)	1 (1.4)	2 (5.4)	0.278
Hypertension, n (%)	43 (62.3)	35 (94.6)	**<0.001**
Hyperuricemia, n (%)	2 (2.9)	4 (10.8)	0.180
SBP (mmHg)	127.34 ± 13.72	126.91 ± 11.35	0.876
DBP (mmHg)	82.10 ± 10.30	81.15 ± 9.67	0.653
ESR (mm/h)	16.92 ± 18.73	21.94 ± 18.04	0.359
Neutrophil–lymphocyte ratio	1.80 ± 0.90	1.88 ± 0.72	0.662
Platelet–lymphocyte ratio	126.29 ± 43.00	128.67 ± 42.91	0.789
Serum albumin (g/dL)	4.33 ± 0.32	4.21 ± 0.22	0.039
Hemoglobin (g/dL)	13.43 ± 1.55	12.05 ± 1.48	**<0.001**
Hematocrit (%)	40.57 ± 4.13	37.26 ± 3.97	**<0.001**
eGFR (mL/min/1.73 m^2^)	89.48 ± 16.18	33.80 ± 13.57	**<0.001**
BUN (mg/dL)	14.12 ± 3.40	30.54 ± 14.20	**<0.001**
Serum creatinine (mg/dL)	0.88 ± 0.19	2.13 ± 0.81	**<0.001**
Serum calcium (mg/dL)	9.25 ± 0.33	9.20 ± 0.34	0.454
Serum phosphorus (mg/dL)	3.51 ± 0.51	3.60 ± 0.53	0.395
Serum potassium (mmol/L)	4.25 ± 0.28	4.59 ± 0.41	**<0.001**
Serum sodium (mmol/L)	140.67 ± 1.66	141.27 ± 2.28	0.122

Data are demonstrated as means ± standard deviations for continuous variables and numbers and percentages for categorical variables. Student’s *t*-test was applied for continuous variables with a normal distribution. However, if normality was not satisfied, the Mann–Whitney U test was used. The Chi-square test or Fisher’s exact test was used for categorical variables. Statistical significance was defined by *p* < 0.05, and significant results are shown in bold. BMI, body mass index; BUN, blood urea nitrogen; CKD, chronic kidney disease; DBP, diastolic blood pressure; ESR, erythrocyte sedimentation rate; SBP, systolic blood pressure.

**Table 3:** Correlation between three types of plant-based diet indices (PDIs) and blood pressure, estimated glomerular filtration rate (eGFR), and biochemical parameters of study participants.

	PDI	hPDI	uPDI
SBP (mmHg)	0.0003	−0.088	0.015
DBP (mmHg)	0.134	−0.039	−0.030
ESR (mm/h)	−0.182	−0.237	−0.061
Neutrophil–lymphocyte ratio	−0.130	−0.202 *	0.123
Platelet–lymphocyte ratio	0.035	−0.223 *	0.083
Serum albumin (g/dL)	−0.050	0.007	−0.004
Hemoglobin (g/dL)	−0.054	0.034	−0.058
Hematocrit (%)	−0.066	0.022	−0.008
eGFR (mL/min/1.73 m^2^)	0.179	0.193 *	−0.418 *
BUN (mg/dL)	−0.106	−0.192 *	0.316 *
Serum creatinine (mg/dL)	−0.215 *	−0.273 *	0.413 *
Serum calcium (mg/dL)	−0.024	−0.037	0.020
Serum phosphorus (mg/dL)	−0.0002	−0.040	0.063
Serum potassium (mmol/L)	0.008	−0.014	0.200 *
Serum sodium (mmol/L)	0.085	0.122	0.197 *

Pearson’s correlation coefficient (*r*) is presented to show a correlation between variables. * *p* < 0.05. Significant results are shown in bold. BUN, blood urea nitrogen; DBP, diastolic blood pressure; ESR, erythrocyte sedimentation rate; hPDI, healthful plant-based diet index; SBP, systolic blood pressure; uPDI, unhealthful plant-based diet index.

**Table 4:** Blood pressure, estimated glomerular filtration rate (eGFR), and biochemical parameters of study participants according to the median values of three types of plant-based diet indices (PDIs).

	PDI (Median Score = 54)			hPDI (Median Score = 57)			uPDI (Median Score = 54)		
	Low PDI (n = 50)	High PDI (n = 56)	*p*	Low hPDI (n = 52)	High hPDI (n = 54)	*p*	Low uPDI (n = 48)	High uPDI (n = 58)	*p*
SBP (mmHg)	124.94 ± 8.84	129.13 ± 15.41	0.103	129.08 ± 12.20	125.45 ± 13.43	0.157	125.31 ± 11.66	128.87 ± 13.84	0.166
DBP (mmHg)	80.30 ± 8.12	83.05 ± 11.38	0.169	81.24 ± 9.48	82.28 ± 10.63	0.605	80.65 ± 8.84	82.80 ± 11.01	0.283
ESR (mm/h)	19.83 ± 11.27	17.52 ± 22.55	0.654	21.00 ± 19.92	16.65 ± 17.46	0.397	16.41 ± 17.97	20.92 ± 19.16	0.377
NLR	1.90 ± 0.88	1.77 ± 0.78	0.411	2.08 ± 0.93	1.60 ± 0.65	**0.003**	1.78 ± 0.90	1.87 ± 0.78	0.576
PLR	121.98 ± 38.23	132.12 ± 46.55	0.233	137.92 ± 44.67	116.38 ± 38.25	**0.010**	124.33 ± 46.39	129.47 ± 39.83	0.548
Serum albumin (g/dL)	4.31 ± 0.27	4.27 ± 0.31	0.492	4.28 ± 0.31	4.30 ± 0.27	0.820	4.27 ± 0.27	4.31 ± 0.31	0.494
Hemoglobin (g/dL)	12.94 ± 1.74	12.94 ± 1.59	0.991	13.02 ± 1.46	12.86 ± 1.84	0.629	13.08 ± 1.60	12.83 ± 1.70	0.435
Hematocrit (%)	39.44 ± 4.57	39.37 ± 4.19	0.931	39.66 ± 3.90	39.15 ± 4.78	0.555	39.62 ± 4.17	39.23 ± 4.52	0.646
eGFR (mL/min/1.73 m^2^)	65.22 ± 30.47	74.35 ± 30.57	0.127	62.94 ± 33.20	76.89 ± 26.68	**0.019**	84.09 ± 25.00	58.42 ± 30.33	**<0.001**
BUN (mg/dL)	21.26 ± 12.49	18.59 ± 11.05	0.245	22.88 ± 14.21	16.93 ± 7.89	**0.009**	15.60 ± 6.45	23.36 ± 13.90	**0.001**
Serum creatinine (mg/dL)	1.45 ± 0.83	1.20 ± 0.72	0.104	1.57 ± 0.91	1.07 ± 0.53	**0.001**	0.97 ± 0.37	1.61 ± 0.90	**<0.001**
Serum calcium (mg/dL)	9.24 ± 0.35	9.22 ± 0.31	0.819	9.24 ± 0.35	9.22 ± 0.31	0.847	9.19 ± 0.29	9.27 ± 0.36	0.226
Serum phosphorus (mg/dL)	3.56 ± 0.54	3.53 ± 0.49	0.797	3.54 ± 0.54	3.55 ± 0.49	0.968	3.49 ± 0.45	3.59 ± 0.56	0.285
Serum potassium (mmol/L)	4.36 ± 0.38	4.37 ± 0.36	0.834	4.34 ± 0.38	4.39 ± 0.36	0.555	4.27 ± 0.33	4.45 ± 0.39	**0.011**
Serum sodium (mmol/L)	140.78 ± 2.15	140.96 ± 1.68	0.622	140.58 ± 1.96	141.17 ± 1.83	0.113	140.65 ± 1.87	141.07 ± 1.94	0.258

Data are demonstrated as means ± standard deviations. Student’s *t*-test was used for variables with a normal distribution. However, if normality was not satisfied, the Mann–Whitney U test was used. Statistical significance was considered by *p* < 0.05, and significant results are shown in bold. BUN, blood urea nitrogen; DBP, diastolic blood pressure; ESR, erythrocyte sedimentation rate; hPDI, healthful plant-based diet index; NLR, neutrophil–lymphocyte ratio; PLR, platelet–lymphocyte ratio; SBP, systolic blood pressure; uPDI, unhealthful plant-based diet index.

**Table 5:** Associations of three types of plant-based diet indices (PDIs) with advanced chronic kidney disease.

	PDI (Median Score = 54)		hPDI (Median Score = 57)		uPDI (Median Score = 54)	
	Low PDI (n = 50)	High PDI (n = 56)	Low hPDI (n = 52)	High hPDI (n = 54)	Low uPDI (n = 48)	High uPDI (n = 58)
Model 1 Odds ratio (95% CI)	1.00 (Ref)	0.654 (0.293–1.459) *p* = 0.300	1.00 (Ref)	**0.256** **(0.109–0.603)** ***p* = 0.002**	1.00 (Ref)	**6.276** **(2.420–16.271)** ***p* < 0.001**
Model 2 Odds ratio (95% CI)	1.00 (Ref)	0.462 (0.185–1.153) *p* = 0.098	1.00 (Ref)	**0.117** **(0.039–0.351)** ***p* < 0.001**	1.00 (Ref)	**8.450** **(2.810–25.409)** ***p* < 0.001**

Odds ratio with 95% confidence interval (CI) for advanced chronic kidney disease unadjusted (model 1) and adjusted for age, sex, and energy intake (model 2) by logistics regression analysis. Statistical significance was defined by *p* < 0.05, and significant results are shown in bold. hPDI, healthful plant-based diet index; uPDI, unhealthful plant-based diet index.

**Table 6:** Dietary intakes of study participants according to the median values of three types of plant-based diet indices (PDIs).

	PDI (Median Score = 54)			hPDI (Median Score = 57)			uPDI (Median Score = 54)		
	Low PDI (n = 50)	High PDI (n = 56)	*p*	Low hPDI (n = 52)	High hPDI (n = 54)	*p*	Low uPDI (n = 48)	High uPDI (n = 58)	*p*
Energy (kcal)	1791.74 ± 435.05	1883.89 ± 494.47	0.313	1912.23 ± 490.23	1771.28 ± 437.90	0.121	1888.69 ± 543.46	1800.48 ± 394.27	0.336
Carbohydrate (g)	255.91 ± 56.79	285.50 ± 78.82	**0.030**	276.62 ± 70.29	266.66 ± 71.15	0.470	267.29 ± 77.51	275.06 ± 64.74	0.575
Fiber (g)	21.60 ± 8.52	25.51 ± 9.80	**0.032**	20.60 ± 8.89	26.62 ± 8.96	**0.001**	25.69 ± 8.61	21.99 ± 9.73	**0.042**
Protein (g)	70.13 ± 21.29	71.47 ± 21.17	0.747	73.39 ± 23.35	68.38 ± 18.65	0.224	75.97 ± 22.95	66.59 ± 18.66	**0.022**
Animal protein (g)	36.13 ± 17.10	34.31 ± 15.94	0.572	38.70 ± 18.22	31.77 ± 13.86	**0.029**	40.07 ± 17.44	31.11 ± 14.50	**0.005**
Plant protein (g)	32.50 ± 9.09	35.38 ± 10.06	0.126	32.85 ± 9.19	35.15 ± 10.08	0.222	34.58 ± 10.58	33.56 ± 8.93	0.590
Fat (g)	50.13 ± 20.12	47.99 ± 20.08	0.584	52.36 ± 21.35	45.76 ± 18.29	0.090	54.18 ± 22.99	44.71 ± 16.20	**0.015**
Cholesterol (mg)	293.67 ± 151.40	294.44 ± 220.81	0.983	295.27 ± 128.13	292.93 ± 236.62	0.950	309.90 ± 140.30	280.98 ± 223.91	0.439
SFAs (g)	10.48 ± 5.69	10.87 ± 7.74	0.770	11.38 ± 6.65	10.01 ± 6.97	0.301	10.65 ± 5.03	10.71 ± 8.05	0.968
MUFAs (g)	12.41 ± 7.09	12.98 ± 9.52	0.728	13.01 ± 7.84	12.43 ± 9.02	0.724	12.83 ± 5.05	12.62 ± 10.47	0.900
PUFAs (g)	12.31 ± 5.89	13.75 ± 7.08	0.261	12.12 ± 6.44	13.99 ± 6.59	0.143	14.61 ± 7.00	11.79 ± 5.92	**0.027**
ω-6 PUFAs (g)	4.77 ± 4.39	5.08 ± 4.53	0.727	4.43 ± 4.52	5.42 ± 4.36	0.252	5.54 ± 4.71	4.43 ± 4.19	0.201
ω-3 PUFAs (g)	1.04 ± 1.17	1.25 ± 2.14	0.534	0.92 ± 1.19	1.37 ± 2.14	0.185	1.55 ± 2.28	0.82 ± 1.05	**0.031**
Vit A (µg RAE)	418.40 ± 216.31	499.70 ± 405.25	0.208	430.39 ± 321.43	491.16 ± 340.17	0.347	530.11 ± 354.74	404.44 ± 301.18	0.051
Vit D (µg)	3.91 ± 3.64	5.30 ± 9.79	0.343	4.89 ± 9.35	4.41 ± 5.33	0.746	5.65 ± 5.50	3.81 ± 8.85	0.212
Vit E (mg α-TE)	14.98 ± 4.96	18.60 ± 7.51	**0.005**	15.66 ± 5.30	18.08 ± 7.61	0.061	18.87 ± 7.85	15.25 ± 4.98	**0.005**
Vit K (µg)	151.54 ± 122.15	162.10 ± 108.17	0.638	141.76 ± 116.22	171.91 ± 111.99	0.177	158.71 ± 112.81	155.80 ± 116.92	0.897
Vit B_1_ (mg)	1.54 ± 0.50	1.67 ± 0.59	0.206	1.59 ± 0.51	1.63 ± 0.59	0.670	1.74 ± 0.62	1.50 ± 0.47	**0.024**
Vit B_2_ (mg)	1.35 ± 0.45	1.33 ± 0.39	0.781	1.36 ± 0.45	1.32 ± 0.39	0.671	1.51 ± 0.45	1.20 ± 0.33	**<0.001**
Niacin (mg NE)	12.30 ± 4.42	13.57 ± 5.39	0.193	12.68 ± 4.99	13.25 ± 4.99	0.556	14.55 ± 5.38	11.66 ± 4.22	**0.003**
Vit B_6_ (mg)	1.78 ± 1.13	1.90 ± 1.20	0.605	1.68 ± 0.91	1.99 ± 1.35	0.174	2.13 ± 1.36	1.60 ± 0.91	**0.020**
Folate (µg DFE)	417.99 ± 127.67	451.09 ± 155.11	0.236	409.39 ± 141.37	460.60 ± 141.56	0.065	474.61 ± 145.80	403.09 ± 133.60	**0.010**
Vit B_12_ (µg)	8.48 ± 5.81	8.19 ± 5.27	0.788	8.29 ± 6.26	8.36 ± 4.72	0.946	9.33 ± 5.03	7.49 ± 5.78	0.086
Vit C (mg)	96.98 ± 63.33	115.45 ± 72.17	0.167	92.82 ± 64.72	120.15 ± 69.85	**0.039**	116.27 ± 71.83	98.86 ± 65.10	0.194
Calcium (mg)	461.22 ± 226.49	520.54 ± 219.48	0.174	481.35 ± 245.21	503.35 ± 202.66	0.615	535.82 ± 206.30	456.76 ± 232.89	0.070
Phosphorus (mg)	1006.38 ± 300.32	1063.32 ± 327.60	0.355	1027.47 ± 324.48	1045.13 ± 308.06	0.774	1134.14 ± 320.80	955.63 ± 288.18	**0.003**
Sodium (mg)	2925.43 ± 941.94	3308.05 ± 1174.16	0.069	3058.52 ± 1178.10	3194.06 ± 989.55	0.522	3440.21 ± 989.24	2868.83 ± 1097.04	**0.006**
Potassium (mg)	2518.73 ± 930.76	2906.80 ± 1081.18	0.052	2530.87 ± 1059.40	2909.49 ± 968.21	0.057	2974.40 ± 950.56	2516.31 ± 1049.15	**0.022**

Data are expressed as means ± standard deviations. Student’s *t*-test was used for variables with a normal distribution. However, if normality was not satisfied, the Mann–Whitney U test was used. Statistical significance was considered by *p* < 0.05, and significant results are shown in bold. DFE, dietary folate equivalent; hPDI, healthful plant-based diet index; MUFAs, monounsaturated fatty acids; NE, niacin equivalent; PUFAs, polyunsaturated fatty acids; RAE, retinol activity equivalent; SFAs, saturated fatty acids; TE, tocopherol equivalent; uPDI, unhealthful plant-based diet index.

**Table 7:** The frequency of reduced protein intake by the medians of three plant-based diet indices (PDIs).

	PDI (Median Score = 54)		hPDI (Median Score = 57)				uPDI (Median Score = 54)		
	Low PDI (n = 50)	High PDI (n = 56)	*p*	Low hPDI (n = 52)	High hPDI (n = 54)	*p*	Low uPDI (n = 48)	High uPDI (n = 58)	*p*
Protein intake (g/kg BW)									
Normal (≥0.8 g/kg BW)	39 (78.0%)	52 (92.9%)	**0.048**	40 (76.9%)	51 (94.4%)	**0.012**	45 (93.75%)	46 (79.3%)	**0.049**
Reduced (<0.8 g/kg BW)	11 (22.0%)	4 (7.1%)		12 (23.1%)	3 (5.6%)		3 (6.25%)	12 (20.7%)	

Data are expressed as numbers and percentages. Fisher’s exact test was applied for statistical analysis. Statistical significance was considered by *p* < 0.05, and significant results are shown in bold. BW, body weight; hPDI, healthful plant-based diet index; uPDI, unhealthful plant-based diet index.

**Table 8:** Correlation between dietary fiber and protein intakes and blood pressure, estimated glomerular filtration rate (eGFR), and biochemical parameters of study participants.

	Dietary Fiber	Dietary Protein	Dietary Animal Protein	Dietary Plant Protein
SBP (mmHg)	−0.095	−0.021	0.041	−0.105
DBP (mmHg)	0.066	0.017	0.021	0.005
ESR (mm/h)	−0.115	−0.057	0.021	−0.192
Neutrophil–lymphocyte ratio	−0.208 *	−0.101	0.003	−0.197 *
Platelet–lymphocyte ratio	−0.135	−0.140	−0.046	−0.236 *
Serum albumin (g/dL)	0.078	0.121	0.111	0.041
Hemoglobin (g/dL)	0.132	0.165	0.092	0.202 *
Hematocrit (%)	0.126	0.148	0.076	0.191
eGFR (mL/min/1.73 m^2^)	0.200 *	0.246 *	0.216 *	0.118
BUN (mg/dL)	−0.317 *	−0.196 *	−0.104	−0.214 *
Serum creatinine (mg/dL)	−0.336 *	−0.228 *	−0.180	−0.158
Serum calcium (mg/dL)	0.055	−0.043	−0.059	0.023
Serum phosphorus (mg/dL)	−0.029	−0.015	0.003	−0.073
Serum potassium (mmol/L)	−0.047	−0.013	−0.018	−0.001
Serum sodium (mmol/L)	0.046	−0.154	−0.143	−0.067

Pearson’s correlation coefficient (*r*) is presented to show a correlation between variables. * *p* < 0.05. Significant results are shown in bold. BUN, blood urea nitrogen; DBP, diastolic blood pressure; ESR, erythrocyte sedimentation rate; SBP, systolic blood pressure.

## Data Availability

The datasets used and/or analyzed during the current study are available from the corresponding author on reasonable request.

## Supplementary Materials

The following supporting information can be downloaded at: https://www.mdpi.com/article/10.3390/nu16162749/s1, Supplementary Table S1: Associations of dietary fiber and protein intakes with advanced chronic kidney disease (CKD); Table S2: Blood pressure, estimated glomerular filtration rate (eGFR), and biochemical parameters of study participants according to the median values of plant-based diet index (PDI) and protein intake; Table S3: Blood pressure, estimated glomerular filtration rate (eGFR), and biochemical parameters of study participants according to the median values of healthful plant-based diet index (hPDI) and protein intake; Table S4: Blood pressure, estimated glomerular filtration rate (eGFR), and biochemical parameters of study participants according to the median values of unhealthful plant-based diet index (uPDI) and protein intake.
