# Supplementary material for: Compliance with a Healthful Plant-Based Diet Is Associated with Kidney Function in Patients with Autosomal Dominant Polycystic Kidney Disease
Source: Nutrients. 2024 Aug 17;16(16):2749. doi: 10.3390/nu16162749 (PMC11356780; doi:10.3390/nu16162749)
Supplement: Supplementary file 1 [file nutrients-16-02749-s001.zip › nutrients-3151798-supplementary.pdf]

Supplementary Materials

Supplementary Table S1. Associations of dietary fiber and protein intakes with advanced chronic kidney disease (CKD)

	**Dietary fiber**	**Dietary protein**	**Dietary animal protein**	**Dietary plant protein**
Odds ratio (95% CI)	0.962 (0.917-1.009) *p* = 0.113	0.983 (0.963-1.004) *p* = 0.116	0.979 (0.953-1.007) *p* = 0.135	0.988 (0.947-1.031) *p* = 0.582

Odds ratio with 95% confidence interval (CI) for advanced chronic kidney disease (unadjusted) by logistics regression analysis. hPDI, healthful plant-based diet index; uPDI, unhealthful plant-based diet index.

Supplementary Table S2. Blood pressure, estimated glomerular filtration rate (eGFR), and biochemical parameters of study participants according to the median values of plant-based diet index (PDI) and protein intake

	PDI					
	Low PDI (n = 50)			High PDI (n = 56)		
	Protein intake (g/kg BW)					
	**Normal (≥ 0.8 g/kg BW; n = 39)**	**Reduced (< 0.8 g/kg BW; n = 11)**	***p***	**Normal (≥ 0.8 g/kg BW; n = 52)**	**Reduced (< 0.8 g/kg BW; n = 4)**	***p***
SBP (mmHg)	124.86 ± 9.67	125.18 ± 5.67	0.917	129.43 ± 15.74	125.25 ± 11.09	0.606
DBP (mmHg)	80.47 ± 8.15	79.73 ± 8.38	0.793	82.86 ± 11.40	85.50 ± 12.48	0.660
ESR (mm/h)	21.22 ± 11.11	14.80 ± 11.58	0.269	18.59 ± 23.93	10.25 ± 6.40	0.499
Neutrophil lymphocyte ratio	1.87 ± 0.74	2.02 ± 1.31	0.608	1.74 ± 0.80	2.03 ± 0.55	0.482
Platelet lymphocyte ratio	119.52 ± 39.47	130.70 ± 33.71	0.397	131.60 ± 47.81	140.69 ± 16.65	0.746
Serum albumin (g/dL)	4.33 ± 0.28	4.23 ± 0.21	0.246	4.27 ± 0.32	4.34 ± 0.27	0.644
Hemoglobin (g/dL)	12.83 ± 1.83	13.34 ± 1.39	0.397	13.01 ± 1.63	12.13 ± 0.77	0.290
Hematocrit (%)	39.11 ± 4.66	40.64 ± 4.22	0.332	39.51 ± 4.29	37.53 ± 2.17	0.366
eGFR (mL/min/1.73 m^2)	65.72 ± 29.73	63.48 ± 34.44	0.832	76.78 ± 29.32	42.76 ± 33.03	**0.031**
BUN (mg/dL)	21.13 ± 12.31	21.73 ± 13.69	0.890	18.00 ± 10.83	26.25 ± 12.61	0.152
Serum creatinine (mg/dL)	1.39 ± 0.80	1.65 ± 0.94	0.355	1.12 ± 0.60	2.23 ± 1.30	**0.002**
Serum calcium (mg/dL)	9.22 ± 0.32	9.32 ± 0.46	0.397	9.20 ± 0.29	9.53 ± 0.46	**0.043**
Serum phosphorus (mg/dL)	3.59 ± 0.52	3.43 ± 0.62	0.372	3.53 ± 0.49	3.63 ± 0.51	0.698
Serum potassium (mmol/L)	4.41 ± 0.41	4.18 ± 0.21	0.084	4.37 ± 0.37	4.43 ± 0.25	0.770
Serum sodium (mmol/L)	140.72 ± 2.05	141.00 ± 2.57	0.705	140.79 ± 1.59	143.25 ± 1.26	**0.004**

Data are expressed as means ± standard deviations. Student's *t*-test was used for variables with a normal distribution. However, if normality was not satisfied, the Mann-Whitney U test was used. Statistical significance was defined by $p < 0.05$, and significant results are shown in bold. BUN, blood urea nitrogen; BW, body weight; DBP, diastolic blood pressure; ESR, erythrocyte sedimentation rate; SBP, systolic blood pressure.

Supplementary Table S3. Blood pressure, estimated glomerular filtration rate (eGFR), and biochemical parameters of study participants according to the median values of healthful plant-based diet index (hPDI) and protein intake

	**hPDI**					
	**Low hPDI (n = 52)**			**High hPDI (n = 54)**		
	**Protein intake (g/kg BW)**					
	**Normal (≥ 0.8 g/kg BW; n = 40)**	**Reduced (< 0.8 g/kg BW; n = 12)**	***p***	**Normal (≥ 0.8 g/kg BW; n = 51)**	**Reduced (< 0.8 g/kg BW; n = 3)**	***p***
SBP (mmHg)	130.57 ± 13.34	124.50 ± 6.08	0.136	125.30 ± 13.63	128.00 ± 11.27	0.739
DBP (mmHg)	81.86 ± 9.89	79.33 ± 8.15	0.427	81.88 ± 10.52	89.00 ± 12.49	0.264
ESR (mm/h)	24.24 ± 21.46	11.83 ± 11.67	0.196	16.86 ±1838	14.67 ± 2.08	0.840
Neutrophil lymphocyte ratio	2.05 ± 0.84	2.15 ± 1.22	0.759	1.60 ± 0.66	1.54 ± 0.60	0.882
Platelet lymphocyte ratio	139.01 ± 48.02	134.38 ± 32.95	0.757	116.08 ± 39.01	123.60 ± 2.42	0.788
Serum albumin (g/dL)	4.29 ± 0.33	4.28 ± 0.25	0.924	4.30 ± 0.28	4.19 ± 0.10	0.490
Hemoglobin (g/dL)	12.96 ± 1.49	13.22 ± 1.40	0.601	12.90 ± 1.88	12.20 ±0.79	0.525
Hematocrit (%)	39.49 ± 3.85	40.22 ± 4.19	0.577	39.21 ± 4.88	38.17 ± 2.92	0.717
eGFR (mL/min/1.73 m^2)	64.37 ± 32.69	58.18 ± 35.93	0.576	78.05 ± 26.19	57.06 ± 33.08	0.188
BUN (mg/dL)	22.83 ± 14.56	23.08 ± 13.61	0.957	16.61 ± 7.53	22.33 ± 13.65	0.225
Serum creatinine (mg/dL)	1.48 ± 0.83	1.88 ± 1.12	0.185	1.05 ± 0.51	1.53 ± 0.67	0.124
Serum calcium (mg/dL)	9.22 ± 0.32	9.28 ± 0.45	0.607	9.19 ± 0.28	9.73 ± 0.23	**0.002**
Serum phosphorus (mg/dL)	3.61 ± 0.53	3.33 ± 0.53	0.112	3.51 ± 0.48	4.10 ± 0.35	**0.045**
Serum potassium (mmol/L)	4.38 ± 0.41	4.24 ± 0.24	0.291	4.39 ± 0.37	4.27 ± 0.32	0.560
Serum sodium (mmol/L)	140.43 ± 1.81	141.08 ± 2.43	0.313	141.02 ± 1.75	143.67 ± 1.53	**0.013**

Data are expressed as means ± standard deviations. Student's *t*-test was used for variables with a normal distribution. However, if normality was not satisfied, the Mann-Whitney U test was used. Statistical significance was defined by $p < 0.05$, and significant results are shown in bold. BUN, blood urea nitrogen; BW, body weight; DBP, diastolic blood pressure; ESR, erythrocyte sedimentation rate; SBP, systolic blood pressure.

Supplementary Table S4. Blood pressure, estimated glomerular filtration rate (eGFR), and biochemical parameters of study participants according to the median values of unhealthful plant-based diet index (uPDI) and protein intake

	**uPDI**					
	**Low uPDI (n = 48)**			**High uPDI (n = 58)**		
	**Protein intake (g/kg BW)**					
	**Normal (≥ 0.8 g/kg BW; n = 45)**	**Reduced (< 0.8 g/kg BW; n = 3)**	***p***	**Normal (≥ 0.8 g/kg BW; n = 46)**	**Reduced (< 0.8 g/kg BW; n = 12)**	***p***
SBP (mmHg)	124.98 ± 11.79	130.33 ± 9.71	0.447	130.29 ± 15.12	123.92 ± 6.05	0.162
DBP (mmHg)	80.58 ± 8.46	81.67 ± 16.17	0.839	83.26 ± 11.73	81.17 ± 8.22	0.566
ESR (mm/h)	16.48 ± 18.64	15.50 ± 2.12	0.942	24.39 ± 20.80	12.00 ± 10.66	0.150
Neutrophil lymphocyte ratio	1.70 ± 0.77	3.02 ± 1.83	**0.012**	1.90 ± 0.78	1.78 ± 0.84	0.643
Platelet lymphocyte ratio	122.52 ± 46.70	150.26 ± 39.30	0.322	129.81 ± 42.45	128.09 ± 28.11	0.899
Serum albumin (g/dL)	4.28 ± 0.28	4.15 ± 0.15	0.461	4.31 ± 0.32	4.28 ± 0.24	0.773
Hemoglobin (g/dL)	13.10 ± 1.58	12.87 ± 2.29	0.812	12.77 ± 1.83	13.05 ± 1.16	0.616
Hematocrit (%)	39.62 ± 4.07	39.63 ± 6.64	0.996	39.06 ± 4.78	39.85 ± 3.47	0.596
eGFR (mL/min/1.73 m^2)	84.79 ± 23.81	73.64 ± 45.13	0.460	59.57 ± 30.09	54.03 ± 32.18	0.578
BUN (mg/dL)	15.31 ± 5.65	20.00 ± 15.59	0.226	23.28 ± 14.23	23.67 ± 13.12	0.933
Serum creatinine (mg/dL)	0.94 ± 0.32	1.34 ± 0.83	0.071	1.52 ± 0.85	1.93 ± 1.08	0.172
Serum calcium (mg/dL)	9.18 ± 0.28	9.30 ± 0.52	0.495	9.23 ± 0.32	9.39 ± 0.46	0.171
Serum phosphorus (mg/dL)	3.46 ± 0.45	3.90 ± 0.36	0.102	3.65 ± 0.54	3.38 ± 0.59	0.130
Serum potassium (mmol/L)	4.27 ± 0.33	4.20 ± 0.26	0.721	4.50 ± 0.40	4.26 ± 0.25	0.054
Serum sodium (mmol/L)	140.51 ± 1.80	142.67 ± 2.08	0.053	141.00 ± 1.76	141.33 ± 2.57	0.600

Data are expressed as means ± standard deviations. Student's *t*-test was used for variables with a normal distribution. However, if normality was not satisfied, the Mann-Whitney U test was used. Statistical significance was defined by $p < 0.05$, and significant results are shown in bold. BUN, blood urea nitrogen; BW, body weight; DBP, diastolic blood pressure; ESR, erythrocyte sedimentation rate; SBP, systolic blood pressure.
